# Neuropathological characterization of Lemur tyrosine kinase 2 (LMTK2) in Alzheimer’s disease and neocortical Lewy body disease

**DOI:** 10.1038/s41598-019-53638-9

**Published:** 2019-11-20

**Authors:** János Bencze, Máté Szarka, Viktor Bencs, Renáta Nóra Szabó, Máté Smajda, Dag Aarsland, Tibor Hortobágyi

**Affiliations:** 1 Department of Pathology, Faculty of Medicine, University of Debrecen, Debrecen, Hungary; 2 MTA-DE Cerebrovascular and Neurodegenerative Research Group, Department of Neurology, University of Debrecen, Debrecen, Hungary; 3 Horvath Csaba Memorial Institute of Bioanalytical Research, Research Centre for Molecular Medicine, University of Debrecen, Debrecen, Hungary; 4 Vitrolink Ltd., Debrecen, Hungary; 5 Institute of Pathology, Faculty of Medicine, University of Szeged, Szeged, Hungary; 6 Department of Old Age Psychiatry, Institute of Psychiatry Psychology and Neuroscience, King’s College London, London, UK; 7 Centre for Age-Related Medicine, SESAM, Stavanger University Hospital, Stavanger, Norway

**Keywords:** Molecular neuroscience, Alzheimer's disease

## Abstract

Alzheimer’s disease (AD) and neocortical Lewy body disease (LBD) are the most common neurodegenerative dementias, with no available curative treatment. Elucidating pathomechanism and identifying novel therapeutic targets are of paramount importance. Lemur tyrosine kinase 2 (LMTK2) is involved in several physiological and pathological cellular processes. Herewith a neuropathological characterization is presented in AD and neocortical LBD samples using chromogenic and fluorescent LMTK2 immunohistochemistry on post-mortem brain tissues and compared them to age-matched controls (CNTs). LMTK2 immunopositivity was limited to the neuronal cytoplasm. Neurons, including tau-positive tangle-bearing ones, showed decreased chromogenic and immunofluorescent labelling in AD in every cortical layer compared to CNT and neocortical LBD. Digital image analysis was performed to measure the average immunopositivity of groups. Mean grey values were calculated for each group after measuring the grey scale LMTK2 signal intensity of each individual neuron. There was significant difference between the mean grey values of CNT vs. AD and neocortical LBD vs. AD. The moderate decrease in neocortical LBD suggests the effect of coexisting AD pathology. We provide neuropathological evidence on decreased neuronal LMTK2 immunolabelling in AD, with implications for pathogenesis.

## Introduction

Dementias are challenging health issues in our aging society. Alzheimer’s disease (AD) and neocortical Lewy body disease (LBD) are the most common causes of dementia accounting for 75–80 percent of the cases^1^. In order to develop more efficient disease-modifying treatments, the molecular, biochemical and genetic alterations (‘omics’) should be better explored^2–4^. Neuropathological hallmarks of AD are the deposition of extracellular β-amyloid plaques and intracellularly aggregated neurofibrillary tangles (NFTs)^5^. Although, neocortical LBD is an α-synucleinopathy characterized by the pathological aggregation and intracellular deposition of α-synuclein forming Lewy bodies and Lewy neurites, coexisting AD-type NFT pathology is also frequently observed. The major component of NFT is hyperphosphorylated microtubule-associated protein tau. Tau is involved in a myriad of physiological cellular functions such as stabilization of microtubules, axonal transport, synaptic transmission or activation of unfolded protein response^6–9^. Under pathological circumstances the hyperphosphorylated tau^10^ is prone to self-aggregation and toxic through sequestering normal tau and other microtubule associated proteins resulting in microtubule disassembly^11^. NFT burden inversely correlates with the number of surviving neurons, suggesting that neurofibrillary lesions have key role in degenerative changes and apoptotic cell loss^12^. Despite the crucial role of tau hyperphosphorylation in the pathogenesis the initiative event leading to pathological activity of numerous kinases remains to be elucidated. Cyclin-dependent kinase 5 (CDK5) and Glycogen synthase kinase-3β (GSK3β) are two major tau-kinases. Moreover, CDK5 can suppress the activity of GSK3β indirectly, but the precise mechanism behind this observation was unknown until Manser *et al*. identified the Lemur tyrosine kinase 2 (LMTK2) as the missing link of the signalling pathway between the two above mentioned tau-kinases^13^. LMTK2 is a member of the membrane-anchored protein kinase family^14^. It has important interacting partners such as CDK5/p35 complex, catalytic subunit of Protein Phosphatase 1 (PP1C), cystic fibrosis transmembrane conductance regulator (CFTR) and myosin VI. Owing to the large LMTK2 interactome it is not surprising that the protein is implicated in several vital physiological functions and diseases (Table 1) including AD^15^. However, human neuropathological studies are not yet available. To fill this gap, in this study chromogenic and fluorescent immunohistochemical (IHC) characterization of LMTK2 was performed on human AD and neocortical LBD brain samples. Digital image analysis was applied to measure the average immunolabelling of experimental groups.

**Table 1:** Implications of Lemur tyrosine kinase 2 (LMTK2) in physiological and pathological processes.

Physiological processes	Pathological conditions
Neuronal differentiation^65^	Prostate cancer^57–60,66^
Intracellular vesicle trafficking^21^	Lung cancer^61,63^
Axonal transport^13^	Cystic fibrosis^20,67^
Spermatogenesis^68^	Neurodegeneration^15^

## Materials and Methods

All procedures were conducted under the ethical approval of the Institutional Ethics Committee of the MRC London Neurodegenerative Diseases Brain Bank (18/WA/0206) at the Institute of Psychiatry Psychology and Neuroscience, King’s College London and the Brains for Dementia Research (BDR) Project (08/H0704/128 + 5). Informed consent for autopsy, neuropathological assessment and research participation were obtained from all subjects and data was anonymised. Block taking for histological and immunohistochemical studies and neuropathological assessment for neurodegenerative diseases was carried out in accordance with standard criteria.

Three experimental groups were established. The observed brain region was the middle frontal gyrus (Brodmann area 9). In the disease groups we selected 6–6 AD and neocortical LBD cases with severe neurodegenerative changes (Braak stage VI and diffuse neocortical, respectively). In these cases dementia was diagnosed in life and confirmed by neuropathology, while in the control (CNT) group there were 6 samples of aged-matched patients (died of non-neurological causes), without significant neurodegenerative changes. There were 11 females and 7 males similarly distributed in the experimental groups. All patients were older than 60-year-old, the average post-mortem delays were 38 (±11.8 SEM) hours for AD, 47.1 (±8.6 SEM) hours for CNT and 43.3 (±7.8 SEM) hours for neocortical LBD cases (Supplementary Table S1).

### Chromogenic immunohistochemistry (CHR-IHC)

7µm-thick formalin-fixed paraffin-embedded (FFPE) sections were routinely de-waxed in decreasing alcohol concentrations (2 × 5 mins. xylene, 5 mins. absolute alcohol, 5 mins. 96% (v/v) alcohol, 5 mins. 80% (v/v) alcohol, 5 mins. 70% (v/v) alcohol), blocked for endogenic peroxidase activity in methanol containing 3% (v/v) H_2_O_2_, and heat-treated in Tris(hydroxymethyl)aminomethane - Ethylenediaminetetraacetic acid (TRIS-EDTA; 10 mM TRIS Base, 1 mM EDTA, pH = 9.0) antigen retrieval buffer solutions using a microwave oven (5 mins. 800 watt, 2 × 5 mins. 250 watt). Non-specific antibody binding sites were blocked with 20 mMol TRIS buffered saline (Sigma-Aldrich, TRIS Buffered Saline 20 × solution, 20 mMol Tris, pH = 7.4) containing 10% (v/v) normal goat serum (Agilent/Dako). Sections were incubated with the primary LMTK2 antibody (KPI-2, clone H-9, SantaCruz Biotechnology) at 4 °C overnight. On the following day samples were incubated for an hour at room temperature with goat anti-mouse biotinylated secondary antibody (Agilent/Dako). Immunohistochemical detection was performed with VECTASTAIN® Elite® ABC HRP Kit (Vector laboratories) – prepared and used according to the manufacturer’s protocol. Chromogen substrate 3,3′-diaminobenzidine tetrahydrochloride (DAB) reagent (Sigma-Aldrich 10 mg tablets) was applied for 4 minutes, then nuclear staining was carried out with Harris’ haematoxylin (30 seconds). Sections were dehydrated in increasing alcohol concentrations (5 mins. 70% (v/v) alcohol, 5 mins. 80% (v/v) alcohol, 5 mins. 96% (v/v) alcohol, 5 mins. absolute alcohol, 5 mins. xylene), then mounted and coverslipped using ClearVue™ Coverslipper (Thermo Fisher Scientific). Dilutions for primary and secondary antibodies were 1:50 and 1:200, respectively in 20 mMol TRIS buffered saline. Working solution for rinsing after certain steps (antigen retrieval, primary and secondary antibodies, ABC kit) was 20 mMol TRIS buffered saline. For quality control purposes IHC ‘negative’ slides without application of primary antibody were also made.

### Immunofluorescent immunohistochemistry (IF-IHC)

Several steps overlap with the above detailed CHR-IHC protocol (de-wax, antigen retrieval, primary antibody), while others are unnecessary for IF-IHC reaction (endogenous peroxidase blocking, biotinylated secondary antibody, chromogen substrate). The working solution was phosphate buffered saline (Biocare Medical, PBS Plus 10x solution, pH = 7.3). Sections were incubated with fluorescent-dye conjugated secondary antibody (Goat Anti-Mouse IgG H&L - Alexa Fluor® 594, Abcam) for an hour at room temperature. Dilution for secondary antibody was 1:200 in working solution. Vector® TrueVIEW™ Autofluorescence Quenching Kit (Vector laboratories) was used to remove non-specific autofluorescence. Coverslipping and nuclear staining were carried out manually using Vectashield® Antifade Mounting Medium with DAPI (4′,6-diamidino-2-phenylindole; Vector laboratories).

### Assessement of CHR-IHC

Slides were scanned with Virtual Slide Microscope VS120 (Olympus Corp.) with the same illumination intensities, exposure times, and camera settings. Cell-specificity, subcellular localisation and association of LMTK2 with disease-specific pathological changes (β-amyloid plaques and NFTs) were evaluated by neuropathologist (TH).

### Digital analysis for IF-IHC

Immunofluorescent microscopy was performed with an Axio Imager Z2 microscope equipped with 20×/0,5 420350–9900 EC Plan-Neofluar objective, Coolcube 1 m S/N: 003274. 60N-C 1″ 1,0×. 426114 camera and appropriate filter sets (Carl Zeiss AG). For digital analysis we used Isis fluorescence imaging platform (MetaSystems Hard & Software GmbH). 5 photos/cases were taken randomly at medium magnification (20×) with the same illumination intensities, exposure times, and camera settings. Data processing was carried out with ImageJ software. Images (90 pcs) were opened in one stack to guarantee that the applied modifications are performed on every single image at the same time with the same settings. After setting the threshold to 1–255 an erode function was executed on the binary images to filter the edges of neurons. The ‘Analyse particles’ module measured the mean greyscale values of the identified objects. To ensure that every assessed particle was neuron, different filters were used: 1) automatic selection based on the size; 2) subsequent manual selection applied to each particle to remove objects which were identified by the software and were not neurons (e.g. blood vessels). The mean greyscale intensities were determined for each case by summarizing the results of individual cells. Then mean grey intensities for CNT, AD and neocortical LBD groups were also calculated.

### Statistical analysis

For statistical analysis of grey intensities of fluorescent IHC signals SigmaPlot 12.0 software (Systat Software Inc.) was used. Shapiro-Wilk normality test, equal variance test, one-way analysis of variance (ANOVA) and all pairwise comparison (Holm-Sidak method) were performed to determine statistical significance of the results. Moreover, analysis of covariance (ANCOVA) was also run in order to evaluate the effects of age at death or post-mortem delay on the results. Testing was carried out with SPSS 25 software (IBM Corp.) dependent variables were IF-IHC results, fixed factors were disease and gender and covariates were age at death and post-mortem delay.

## Results

**Figure 1:**
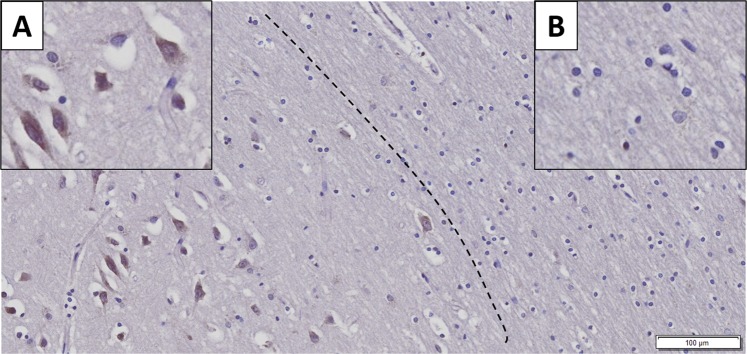
Neuron-specificity of lemur tyrosine kinase 2 (LMTK2) in human brain tissue. The figure depicts the border (dashed line) between grey (on the left) and white (on the right) matter. Neurons show cytoplasmic immunopositivity (Insert **A**), while in the glial cells positive reaction is not detectable (Insert **B**). [The protein was visualized by 3,3′-Diaminobenzidine (DAB) chromogen. Nuclear counterstain was haematoxylin. Scale bar: 100 µm].

**Figure 2:**
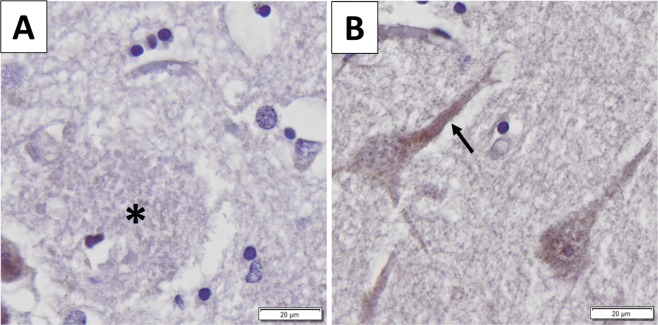
Association between lemur tyrosine kinase 2 (LMKT2) and Alzheimer’s disease (AD)-specific pathological changes. Panel (A) There is no LMTK2 immunoreaction in β-amyloid plaque (black star). Panel (B) The average LMTK2 immunopositivity is similarly weak in tangle-bearing (on the left) and morphologically normal (on the right) neurons . However, neurofibrillary tangles show slightly stronger reaction (black arrow). [The protein was visualized by 3,3′-Diaminobenzidine (DAB) chromogen. Nuclear counterstain  with haematoxylin. Scale bar: 20 µm].

**Figure 3:**
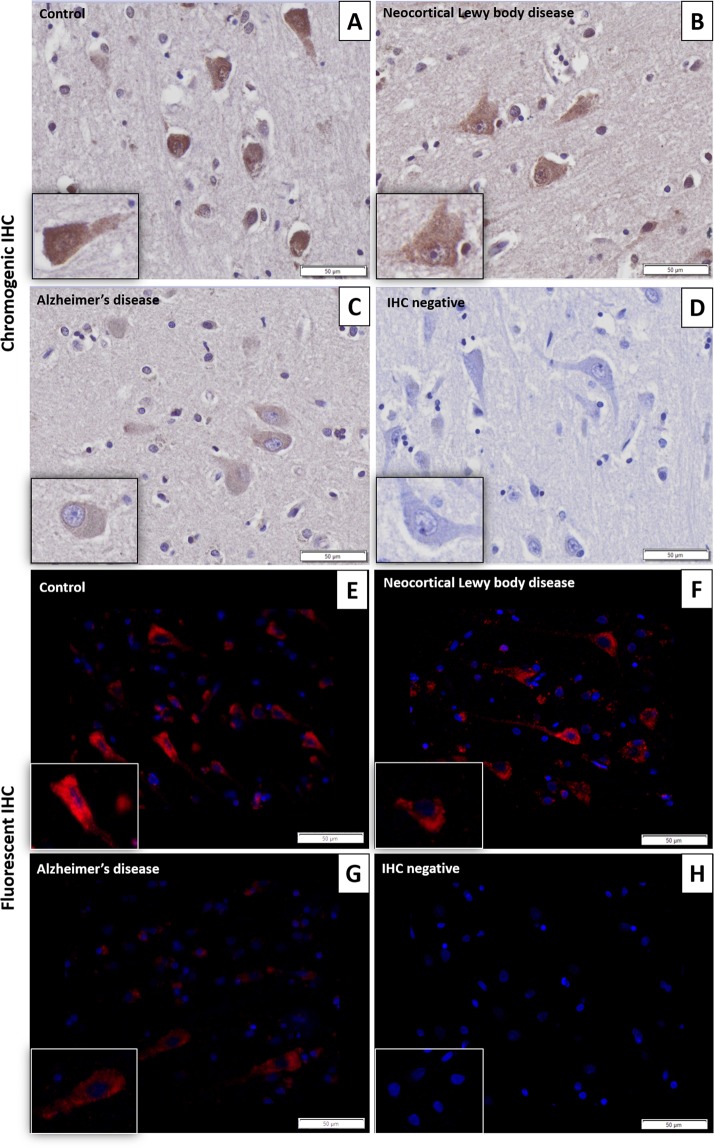
Decreased labelling of lemur tyrosine kinase 2 (LMTK2) in Alzheimer’s disease (AD) as compared to neocortical Lewy body disease (LBD) and control brains. In the control and neocortical LBD the LMTK2 immunopositivity is more intense with chromogenic (brown)(Panels A,B) and fluorescent (red) technique (Panels E,F), respectively, compared to AD (Panels C,G). IHC negative slides (without application of primary anti-LMTK2 antibody) are also presented (Panels D,H). [The protein was visualized by 3,3′-Diaminobenzidine (DAB) chromogen and Alexa Fluor® 594 fluorescent dye. Nuclear counterstains were haematoxylin and 4′,6-diamidino-2-phenylindole (DAPI) on chromogenic and fluorescent slides, respectively. Scale bar: 50 µm].

**Figure 4:**
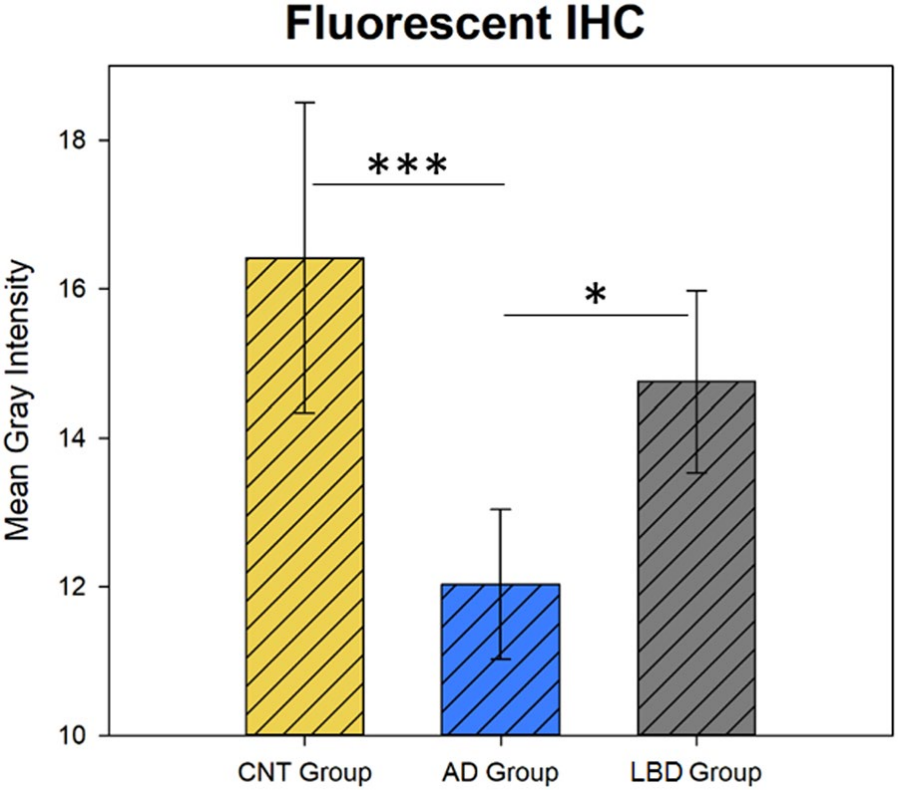
Fluorescent lemur tyrosine kinase 2 (LMTK2) immunohistochemistry (IHC) intensity plots of control (CNT), Alzheimer’s disease (AD) and neocortical Lewy body disease (LBD) groups. The mean grey intensities of the fluorescent signals are presented. There are statistically significant differences between CNT-AD (p < 0.001(***)) and neocortical LBD- AD (p < 0.05 (*)), while the comparison between CNT and neocortical LBD groups does not show significant result. [Color code: yellow columns = CNT; blue columns = AD, grey columns = LBD].

Immunohistochemistry showed strong neuron-specific cytoplasmic reaction; glial cells and nuclei of neurons were negative (Fig. 1). In AD the extracellular β-amyloid plaques did not display positive reaction (Fig. 2. Panel A). The average LMTK2 immunopositivity was similarly weak in both tangle-bearing neurons and morphologically normal cells. However, NFTs showed slightly stronger reaction (Fig. 2. Panel B). The difference between the immunopositivity of the experimental groups was perceptible to the naked eye (Fig. 3). Basically, the LMTK2 immunoreaction in AD was weaker compared to CNT and neocortical LBD. This tendency was detectable in every cortical layer (Supplementary Fig. S1). Number of analysed neurons were 1272 on fluorescent images. Figure 4 depicts the summarized mean grey intensities of CNT, AD and neocortical LBD groups. Although the mean intensity values of individual cases vary in the experimental groups (ranged between 13.6–19, 10.8–13.4 and 12.8–16.4 in CNT, AD and neocortical LBD, respectively), their standard deviations tend to be almost identical providing adequate basis for statistical analysis. One-way ANOVA showed statistically significant difference among the mean grey intensities of CNT, AD and neocortical LBD groups with fluorescent (p < 0,001) IHC. All pairwise comparison revealed statistically significant alteration between CNT-AD (p < 0.001) and neocortical LBD-AD (p = 0.014) groups (Fig. 4). There was no significant result in the case of CNT versus (vs) neocortical LBD comparison. According to ANCOVA test neither the age at death (p = 0.100), nor the post-mortem delay (p = 0.718) influenced significantly the results of IF-IHC.

## Discussion

In accordance with the literature we found that in neural tissue LMTK2 expression was localized to the cytoplasm of neurons, while nuclei and glial cells were negative^16^. β-amyloid plaques in AD did not cumulate LMTK2 proposing that it is not involved in the formation of these protein aggregates. Interestingly, in AD tangle-bearing neurons and morphologically normal cells showed similarly weak average cytoplasmic immunoreaction suggesting that LMTK2 reduction is a general feature in the NFT-affected brain regions. This alteration is probably an early event of the pathogenesis which precedes and predicts the appearance of morphological changes. Contrarily, NFTs themselves displayed slightly stronger LMTK2 positivity. It could be explained by the followings: on the one hand LMTK2 may be a currently undescribed constituent of NFTs and on the other hand NFT as a condense protein aggregate may cumulate LMTK2 even though the average cytoplasmic expression of the enzyme is relatively low in tangle-bearing neurons. In order to evaluate the immunofluorescent LMTK2 immunoreaction we used a slightly modified, adapted version of a previously described ImageJ software-based method^17^. In our study we demonstrated decreased LMTK2 immunopositivity in AD samples compared to neocortical LBD and age-matched CNT. Statistical tests showed significant difference between the mean grey intensities of CNT-AD and neocortical LBD-AD groups, while in the case of CNT- neocortical LBD comparison, in spite of the decreased immunopositivity in LBD, the results were not statistically significant. The moderately reduced average immunopositivity in neocortical LBD compared to CNT may resulted from the frequent coexistence of a AD-type pathology^18^. This observation is suggesting that decreased LMTK2 expression is probably an AD- (or tauopathy-) specific alteration and it may not associate directly to α-synucleinopathies. In accordance with our results a recent genome-wide gene-expression study has detected decreasing LMTK2 expression during disease progression in the cortical and hippocampal regions of a transgenic tau (Tau P301L) mouse model [43]. Furthermore, in a complementary work *Morotz et al*. have recently found reduced LMTK2 levels in human AD samples by western blot analysis^19^. Since they investigated many of our patients’ frozen tissue samples, their study validates our results. However, they could not provide neither morphological nor LBD analysis. Consequently, our study provides the first neuropathological characterization of LMTK2 in post-mortem human AD and neocortical LBD tissue.

Although the analysis of LMTK2 signalling is out of the scope of this current work, based on recent cell biological and animal model studies reduced LMTK2 level alters three important cellular mechanisms which may contribute to neurodegeneration: (i) axonal transport, (ii) tau hyperphosphorylation, (iii) apoptosis^15^ (Supplementary Fig. S2).

### Disrupted axonal transport

As polarized cells, neurons require harmonized axonal transport to supply their dendrites and axon(s) with essential macromolecules produced by the soma. Under physiological conditions LMTK2 is involved in the coordination of intracellular molecular trafficking^20,21^. However, any defect of this well-organized machinery may result in the pathological accumulation of intracellular organelles and proteins. It is known that disrupted axonal transport is an early event in the pathogenesis of several neurodegenerative diseases^22^. A recent paper has described an LMTK2-mediated pathway in connection with a Kinesin-1-associated axonal transport^13^. As a major motor protein Kinesin-1 plays a crucial role in the axonal transport of subcellular organelles, intracellular vesicles and signalling proteins^23–25^. The protein has two heavy chains moving along microtubules, and two light chains – responsible for cargo binding. Phosphorylation of Kinesin-1 light chains (KLCs) is a crucial regulatory mechanism^23^. GSK3β can transfer phosphoryl groups to KLC2 directly, resulting in cargo release and repressed Kinesin-1- dependent transport^26–28^. Manser and co-workers have recently described how CDK5 influences this signalling pathway via LMTK2-PP1C interaction^27,29^ CDK5/p35 complex as an upstream activator of LMTK2 phosphorylates it on serine-1418. In this case LMTK2 can phosphorylate the catalytic subunit of PP1 leading to decreased activity of the phosphatase enzyme. Finally, these steps contribute to increased inhibitory phosphorylation on GSK3β serine-9. Since, supressed GSK3β is not able to inhibit KLC2 this signalling pathway supports cargo binding to the subunit^13,29^. One of the macromolecules transported by Kinesin-1 is mothers against decapentaplegic homolog 2 (Smad2)^13,30^. Smad2 is an essential member of transforming growth factor-β (TGF-β) nucleocytoplasmic signalling pathway^31^. siRNA- induced silencing of LMTK2 disrupts Kinesin-1- mediated axonal transport of Smad2, that in turn inhibits TGF-β nuclear signalling^32^. Disorganised TGF-β/Smad2 pathway has been reported in Alzheimer’s disease^32,33^. These findings conclude that reduced LMTK2 level may alter physiological axonal transport, thereby contribute to neurodegeneration.

### Tau hyperphosphorylation

Tau hyperphosphorylation is a pathological hallmark of AD^34^. Role of CDK5 and GSK3β in this process was investigated in several studies^35–38^. Physiologically, CDK5 is activated by the cofactor p35^39^. Under neuronal stress increased calpain activation results in cleavage of p35 producing p25 and p10^40,41^. CDK5/p25 complex has prolonged half-life/activity leading to hyperphosphorylation of downstream targets (e.g. tau)^38,42–44^. As we previously described CKD5/p35 can attenuate GSK3β indirectly via LMTK2- mediated pathway. However, in the lack of LMTK2 signalling PP1 can remove inhibitory phosphoryl groups from GSK3β^13,29^. Enhanced GSK3β activity is highly implicated in the hyperphosphorylation of tau protein^35^. We suppose that in AD the aberrant coupling of upstream regulator CDK5 with p25 and further currently unknown mechanisms contribute to decreased activity and expression of LMTK2 which leads to overactivation of GSK3β and ultimately to the formation of hyperphosphorylated tau and NFTs.

### Apoptosis

*Conti* *et al*. have recently characterized a potential LMTK2- mediated pathway in the regulation of apoptosis^45^. In their study siRNA- induced LMTK2 silencing reduced the levels of antiapoptotic B-cell lymphoma-2 (Bcl-2) and B-cell lymphoma-extra-large (Bcl-xL), and elevated the level of proapoptotic Bcl-2-interacting mediator of cell death (Bim). These cells showed increased sensitivity to tumour necrosis factor-related apoptosis-inducing ligand (TRAIL) and certain chemotherapeutic agents. Altered Bim and Bcl-2 levels resulted from the missing LMTK2- induced inhibition on PP1C and GSK3β^45^. Furthermore, increased Bim expression has been also observed in AD brain samples, while elevated Bcl-2 level seems to be protective against AD-related cell stressors^46,47^. Group of researchers consider neurodegeneration as the ‘cancer of neurons’^48–50^. Although neurons lost their proliferative capacity, certain harmful stimuli (e.g. excessive oxidative stress, β-amyloid accumulation, etc.) can induce aberrant cell cycle re-entry^51–55^. However, this abnormal process is terminated by different pro-apoptotic mechanisms^54,56^. Presumably, noxious stimuli which trigger uncontrolled proliferation and cancer formation in other tissues could result in extensive neuronal death in the central nervous system. Role of LMTK2 has been discussed in several malignant tumours. Decreased LMTK2 expression was observed in prostate adenocarcinoma as well as in other common malignancies such as lung or skin cancers^57–63^. Since, LMTK2 is involved in the sophisticated regulation of apoptotic factors we could consider it as a tumour suppressor protein. Therefore, downregulation of LMTK2 may contribute to both tumourigenesis and neurodegeneration via dysregulation of neuronal homeostasis.

Strengths of study include providing the first histopathological characterization of LMTK2 in the two most frequent human neurodegenerative dementias. We have described LMTK2’s cell specificity, intracellular localisation, cortical distribution and association with the hallmark neurodegenerative changes (tangle-bearing neurons, β-amyloid plaques). We have demonstrated that LMTK2 immunopositivity does not change remarkably in neocortical LBD compared to CNT patients. In addition, we validated the results of complementary proteomic and cell biological studies confirming that the protein’s level is significantly decreased in AD. The used IF-IHC  is an easily accessible, relatively cheap and fast technique compared to quantitative molecular biological methods (i.e. western blot, qPCR) which are not feasible in the first-line pathological workup.

Limitations of the study: In the current work we have not performed further quantitave analysis (i.e. western blot, qPCR). However, in a complementary work *Morotz et al*. validated the decreased LMTK2 expression on partially the same CNT and AD cases with western blot analysis^19^. Finally, although the number of cases (n = 6/groups) are relatively low, the tendency of immunopositivity within the experimental groups (without notable outliers), the significant difference among AD, neocortical LBD and CNT groups, the large number of analysed neurons and the complementary western blot data further strongly support our findings.

## Conclusion and Prospects

This study provides the first neuropathological characterization of LMTK2 in human AD and neocortical LBD samples. Besides the previously undescribed morphological features, we have detected significantly decreased LMTK2 immunopositivity in AD compared to CNT or neocortical LBD groups. According to our results LMTK2 reduction is specific to AD (or tauopathies), while the moderately decreased immunoreaction in LBD compared to CNT was probably caused by the frequently coexisting AD-type pathology. These are consistent with evidences from cell biological and animal model studies. LMTK2 may contribute to neurodegeneration in AD via three mechanisms: disrupted axonal transport, tau hyperphosphorylation, and enhanced apoptosis. Since available treatments in AD are symptomatic with a limited efficacy, there is a need for discovering novel therapeutic targets. A recent study has identified a 2-O-Tetradecanoylphorbol-13-acetate (TPA) responsive element in the promoter of LMTK2 gene. Stimulation of this region by the synthetic Protein Kinase C activator TPA increased the expression of LMTK2^64^ which is theoretically beneficial in AD. However, further investigations are required to elucidate LMTK2’s role in neurodegeneration and as a therapeutic target in AD.

## Supplementary information

Supplementary material

## Data Availability

The datasets used and/or analysed during the current study available from the corresponding author on reasonable request.

## References

[1] Alzheimer’s Association (2017). Alzheimer’s disease facts and figures. Alzheimer’s Dement..

[2] Whitfield DR (2014). Assessment of ZnT3 and PSD95 protein levels in Lewy body dementias and Alzheimer’s disease: Association with cognitive impairment. Neurobiol. Aging.

[3] Whitfield DR (2014). Depression and synaptic zinc regulation in Alzheimer's disease, dementia with Lewy bodies and Parkinson’s disease dementia. Am. J. Geriatr. Psychiatry.

[4] Bereczki E (2018). Synaptic markers of cognitive decline in neurodegenerative diseases: a proteomic approach. Brain.

[5] Richter, R. W. & Richter, B. Z. Alzheimer’s Disease. (Spinger, 2004).

[6] Guo T, Noble W, Hanger DP (2017). Roles of tau protein in health and disease. Acta Neuropathologica.

[7] Bereczki E (2016). Synaptic proteins predict cognitive decline in Alzheimer’s disease and Lewy body dementia. Alzheimer’s Dement..

[8] Tiwari SS (2015). Evidence that the presynaptic vesicle protein CSPalpha is a key player in synaptic degeneration and protection in Alzheimer’s disease. Mol. Brain.

[9] Baek JH (2016). Unfolded protein response is activated in Lewy body dementias. Neuropathol. Appl. Neurobiol..

[10] Kopke E (1993). Microtubule-associated protein tau. Abnormal phosphorylation of a non- paired helical filament pool in Alzheimer disease. J. Biol. Chem..

[11] Alonso AD, Grundke-Iqbal I, Barra HS, Iqbal K (1997). Abnormal phosphorylation of tau and the mechanism of Alzheimer neurofibrillary degeneration: sequestration of microtubule-associated proteins 1 and 2 and the disassembly of microtubules by the abnormal tau. Proc. Natl. Acad. Sci. USA.

[12] AVILA J (2004). Role of Tau Protein in Both Physiological and Pathological Conditions. Physiol. Rev..

[13] Manser C (2012). Lemur tyrosine kinase-2 signalling regulates kinesin-1 light chain-2 phosphorylation and binding of Smad2 cargo. Oncogene.

[14] Tomomura M (2007). Structural and functional analysis of the apoptosis-associated tyrosine kinase (AATYK) family. Neuroscience.

[15] Bencze J (2018). Biological function of Lemur tyrosine kinase 2 (LMTK2): implications in neurodegeneration. Mol. Brain.

[16] Tissue expression of LMTK2 - Staining in cerebral cortex - The Human Protein Atlas, www.proteinatlas.org/ENSG00000164715-LMTK2/tissue/cerebral+cortex (2019).

[17] Labno, C. Basic Intensity Quantification with ImageJ, www.unige.ch/medecine/bioimaging/files/1914/1208/6000/Quantification.pdf (2019).

[18] McKeith IG (2017). Diagnosis and management of dementia with Lewy bodies. Neurology.

[19] Mórotz GM (2019). LMTK2 binds to kinesin light chains to mediate anterograde axonal transport of cdk5/p35 and LMTK2 levels are reduced in Alzheimer’s disease brains. Acta Neuropathol. Commun..

[20] Chibalina MV, Seaman MNJ, Miller CC, Kendrick-Jones J, Buss F (2007). Myosin VI and its interacting protein LMTK2 regulate tubule formation and transport to the endocytic recycling compartment. J. Cell Sci..

[21] Inoue T (2008). BREK/LMTK2 is a myosin VI-binding protein involved in endosomal membrane trafficking. Genes to Cells.

[22] De Vos KJ, Grierson AJ, Ackerley S, Miller CCJ (2008). Role of Axonal Transport in Neurodegenerative Diseases. Annu. Rev. Neurosci..

[23] Hirokawa N, Niwa S, Tanaka Y (2010). Molecular motors in neurons: Transport mechanisms and roles in brain function, development, and disease. Neuron.

[24] Saxton WM, Hollenbeck PJ (2012). The axonal transport of mitochondria. J. Cell Sci..

[25] Cai Q, Pan P-Y, Sheng Z-H (2007). Syntabulin-Kinesin-1 Family Member 5B-Mediated Axonal Transport Contributes to Activity-Dependent Presynaptic Assembly. J. Neurosci..

[26] Vagnoni A (2012). Calsyntenin-1 mediates axonal transport of the amyloid precursor protein and regulates aβ production. Hum. Mol. Genet..

[27] Morfini G (2002). Glycogen synthase kinase 3 phosphorylates kinesin light chains and negatively regulates kinesin-based motility. EMBO J..

[28] Morfini G (2004). A novel CDK5-dependent pathway for regulating GSK3 activity and kinesin-driven motility in neurons. EMBO J..

[29] Manser C, Vagnoni A, Guillot F, Davies J, Miller CCJ (2012). Cdk5/p35 phosphorylates lemur tyrosine kinase-2 to regulate protein phosphatase-1C phosphorylation and activity. J. Neurochem..

[30] Batut J, Howell M, Hill CS (2007). Kinesin-Mediated Transport of Smad2 Is Required for Signaling in Response to TGF-β Ligands. Dev. Cell.

[31] Hill CS (2009). Nucleocytoplasmic shuttling of Smad proteins. Cell Research.

[32] Katsuno M (2011). Transforming growth factor-β signaling in motor neuron diseases. Curr. Mol. Med..

[33] Town T (2008). Blocking TGF-β-Smad2/3 innate immune signaling mitigates Alzheimer-like pathology. Nat. Med..

[34] Hanger DP (2014). Intracellular and extracellular roles for tau in neurodegenerative disease. Journal of Alzheimer’s Disease.

[35] Pei JJ (1999). Distribution of active glycogen synthase kinase 3β (GSK-3β) in brains staged for Alzheimer disease neurofibrillary changes. J. Neuropathol. Exp. Neurol..

[36] Pei JJ (1998). Accumulation of cyclin-dependent kinase 5 (cdk5) in neurons with early stages of Alzheimer’s disease neurofibrillary degeneration. Brain Res..

[37] Noble W (2003). Cdk5 is a key factor in tau aggregation and tangle formation *in vivo*. Neuron.

[38] Flaherty DB, Soria JP, Tomasiewicz HG, Wood JG (2000). Phosphorylation of human tau protein by microtubule-associated kinases: GSK3β and cdk5 are key participants. J. Neurosci. Res..

[39] Kesavapany S (2003). Identification of a novel, membrane-associated neuronal kinase, cyclin-dependent kinase 5/p35-regulated kinase. J. Neurosci..

[40] Lee MS (2000). Neurotoxicity induces cleavage of p35 to p25 by calpain. Nature.

[41] Nath R (2000). Processing of cdk5 activator p35 to its truncated form (p25) by calpain in acutely injured neuronal cells. Biochem. Biophys. Res. Commun..

[42] Patrick GN (1999). Conversion of p35 to p25 deregulates Cdk5 activity and promotes neurodegeneration. Nature.

[43] Engmann O (2011). Cyclin-Dependent Kinase 5 Activator p25 Is Generated During Memory Formation and Is Reduced at an Early Stage in Alzheimer’s Disease. Biol. Psychiatry.

[44] Engmann O (2011). Schizophrenia is associated with dysregulation of a Cdk5 activator that regulates synaptic protein expression and cognition. Brain.

[45] Conti A (2017). Lemur tyrosine kinase 2 (LMTK2) is a determinant of cell sensitivity to apoptosis by regulating the levels of the BCL2 family members. Cancer Lett..

[46] Engidawork E, Gulesserian T, Seidl R, Cairns N, Lubec G (2001). Expression of apoptosis related proteins in brains of patients with Alzheimer’s disease. Neurosci. Lett..

[47] Guo Q (1997). Alzheimer’s presenilin mutation sensitizes neural cells to apoptosis induced by trophic factor withdrawal and amyloid beta-peptide: involvement of calcium and oxyradicals. J. Neurosci..

[48] Plun-Favreau H, Lewis PA, Hardy J, Martins LM, Wood NW (2010). Cancer and Neurodegeneration: Between the Devil and the Deep Blue Sea. PLOS Genet..

[49] Garcia-Ratés S, Greenfield S (2017). Cancer and neurodegeneration: two sides, same coin?. Oncotarget.

[50] Du L, Pertsemlidis A (2011). Cancer and neurodegenerative disorders: Pathogenic convergence through microRNA regulation. Journal of Molecular Cell Biology.

[51] Monaco EA, Vallano M (2005). Lou. Role of protein kinases in neurodegenerative disease: cyclin-dependent kinases in Alzheimer’s disease. Front. Biosci..

[52] Ahn KW (2008). Swedish amyloid precursor protein mutation increases cell cycle-related proteins *in vitro* and *in vivo*. J. Neurosci. Res..

[53] Giovanni A, Wirtz-Brugger F, Keramaris E, Slack R, Park DS (1999). Involvement of cell cycle elements, cyclin-dependent kinases, pRb, and E2F x DP, in B-amyloid-induced neuronal death. J. Biol. Chem..

[54] Currais A, Hortobágyi T, Soriano S (2009). The neuronal cell cycle as a mechanism of pathogenesis in Alzheimer’s. Aging (Albany. NY)..

[55] Malik B (2008). Loss of neuronal cell cycle control as a mechanism of neurodegeneration in the Presenilin-1 Alzheimer’s disease brain. Cell Cycle.

[56] Liu DX, Greene LA (2001). Neuronal apoptosis at the G1/S cell cycle checkpoint. Cell and Tissue Research.

[57] Shah K, Bradbury NA (2015). Lemur Tyrosine Kinase 2, a novel target in prostate cancer therapy. Oncotarget.

[58] Shah K, Bradbury NA (2015). Kinase Modulation of Androgen Receptor Signaling: Implications for Prostate Cancer. Cancer Cell Microenviron..

[59] Harries LW, Perry JR, McCullagh P, Crundwell M (2010). Alterations in LMTK2, MSMB and HNF1B gene expression are associated with the development of prostate cancer. BMC Cancer.

[60] Eeles RA (2008). Multiple newly identified loci associated with prostate cancer susceptibility. Nat. Genet..

[61] Seo JS (2012). The transcriptional landscape and mutational profile of lung adenocarcinoma. Genome Res..

[62] Feng DD (2015). The associations between Parkinson’s disease and cancer: the plot thickens. Transl. Neurodegener..

[63] Liu X (2016). Next-generation sequencing of pulmonary sarcomatoid carcinoma reveals high frequency of actionable MET gene mutations. J. Clin. Oncol..

[64] Dey I, Bradbury NA (2017). Activation of TPA-response element present in human Lemur Tyrosine Kinase 2 (lmtk2) gene increases its expression. Biochem. Biophys. Reports.

[65] Kawa S, Fujimoto J, Tezuka T, Nakazawa T, Yamamoto T (2004). Involvement of BREK, a serine/threonine kinase enriched in brain, in NGF signalling. Genes to Cells.

[66] Pozo K (2013). The Role of Cdk5 in Neuroendocrine Thyroid Cancer. Cancer Cell.

[67] Luz S (2014). LMTK2-mediated phosphorylation regulates CFTR endocytosis in human airway epithelial cells. J. Biol. Chem..

[68] Kawa S (2006). Azoospermia in mice with targeted disruption of the Brek/Lmtk2 (brain-enriched kinase/lemur tyrosine kinase 2) gene. Proc. Natl. Acad. Sci. USA.

