# Supplementary material for: Neuropathological characterization of Lemur tyrosine kinase 2 (LMTK2) in Alzheimer’s disease and neocortical Lewy body disease
Source: Sci Rep. 2019 Nov 20;9:17222. doi: 10.1038/s41598-019-53638-9 (PMC6868282; doi:10.1038/s41598-019-53638-9)
Supplement: Supplementary file 1 — Supplementary material [file 41598_2019_53638_MOESM1_ESM.docx]

**Supplementary table S1. Human post-mortem samples: age at death, sex, post-mortem delay, brain weight and pH, APOE gene polymorphism, neuropathological Braak tau staging and presence of amyloid angiopathy.**

[AD: Alzheimer’s disease, LBD: Lewy body disease PMD: Post-mortem delay, APOE: Apolipoprotein E, MMSE: Mini-Mental State Examination, MAC: Minimal ageing changes - Braak tau stage ‘+’ (less than Braak stage I)]

|  | **Autopsy N^o^** | **Sex** | **Age  (years)** | **PMD (hours)** | **Brain Weight (grams)** | **Brain pH** | **APOE gene polymorphism** | **Final MMSE** | **Neuropathology Braak tau stage** | **Amyloid angiopathy** |
| --- | --- | --- | --- | --- | --- | --- | --- | --- | --- | --- |
| **Control cases** | A007/15 | F | 74 | 66 | 1191 | 6.53 | - | - | Stage II | - |
|  | A158/14 | F | 73 | 73 | 1095 | 6.4 | - | - | Stage I | - |
|  | A319/14 | F | 90 | 44 | 1150 | 5.79 | - | - | Stage II | - |
|  | A002/13 | M | 90 | 45 | 1252 | 6.9 | - | - | MAC | Mild |
|  | A261/12 | M | 63 | 23 | 1464 | - | - | - | MAC | - |
|  | A308/14 | F | 66 | 78 | 1414 | 5.93 | - | - | MAC | - |
| **Neocortical LBD cases** | A062/16 | M | 86 | 29 | 1234 | - | - | - | - | Moderate |
|  | A274/16 | F | 83 | 52 | 1164 | - | - | - | - | - |
|  | A341/15 | M | 79 | 48 | 1519 | 6.77 | - | - | Stage I  (coexisting) | - |
|  | A276/14 | F | 90 | 67 | 979 | 6.75 | ε3/3 allele | 18 | Stage III  (coexisting) | Mild |
|  | A304/06 | F | 92 | 55 | 1244 | 6.59 | - | - | Stage III  (coexisting) | - |
|  | A040/10 | F | 87 | 9 | 1060 | 6.13 | - | - | Stage II  (coexisting) | - |
| **AD cases** | A092/15 | F | 86 | 13 | 1056 | 6.68 | ε3/4 allele | 0 | Stage VI | Severe |
|  | A192/13 | M | 77 | 71 | 1325 | 6.86 | - | - | Stage VI | Severe |
|  | A277/12 | M | 79 | 20 | 1270 | 6.88 | ε3/4 allele | 16 | Stage VI | Moderate |
|  | A308/13 | F | 74 | 20 | 1053 | - | - | 15 | Stage VI | Mild |
|  | A355/13 | M | 73 | 26 | 1239 | 5.75 | ε3/4 allele | - | Stage VI | Extensive |
|  | A377/14 | F | 85 | 79 | 1135 | - | - | - | Stage VI | Mild |

**Supplementary figure S1. LMTK2 immunopositivity of different cortical layers in Alzheimer’s disease (AD) and control brain samples.**

All of the cortical layers show decreased LMTK2 immunopositivity in AD (Panel A) compared to age-matched control (panel B) with chromogenic immunohistochemistry. [Dashed lines display the approximate border of cortical layers - I: Molecular layer, II: External granular layer, III: External pyramidal layer, IV: Internal granular layer, V: Internal pyramidal layer, VI: Multiform layer. The protein was visualized by 3,3′-Diaminobenzidine (DAB) chromogen. Nuclear counterstain was haematoxylin. Scale bar:100 µm.]


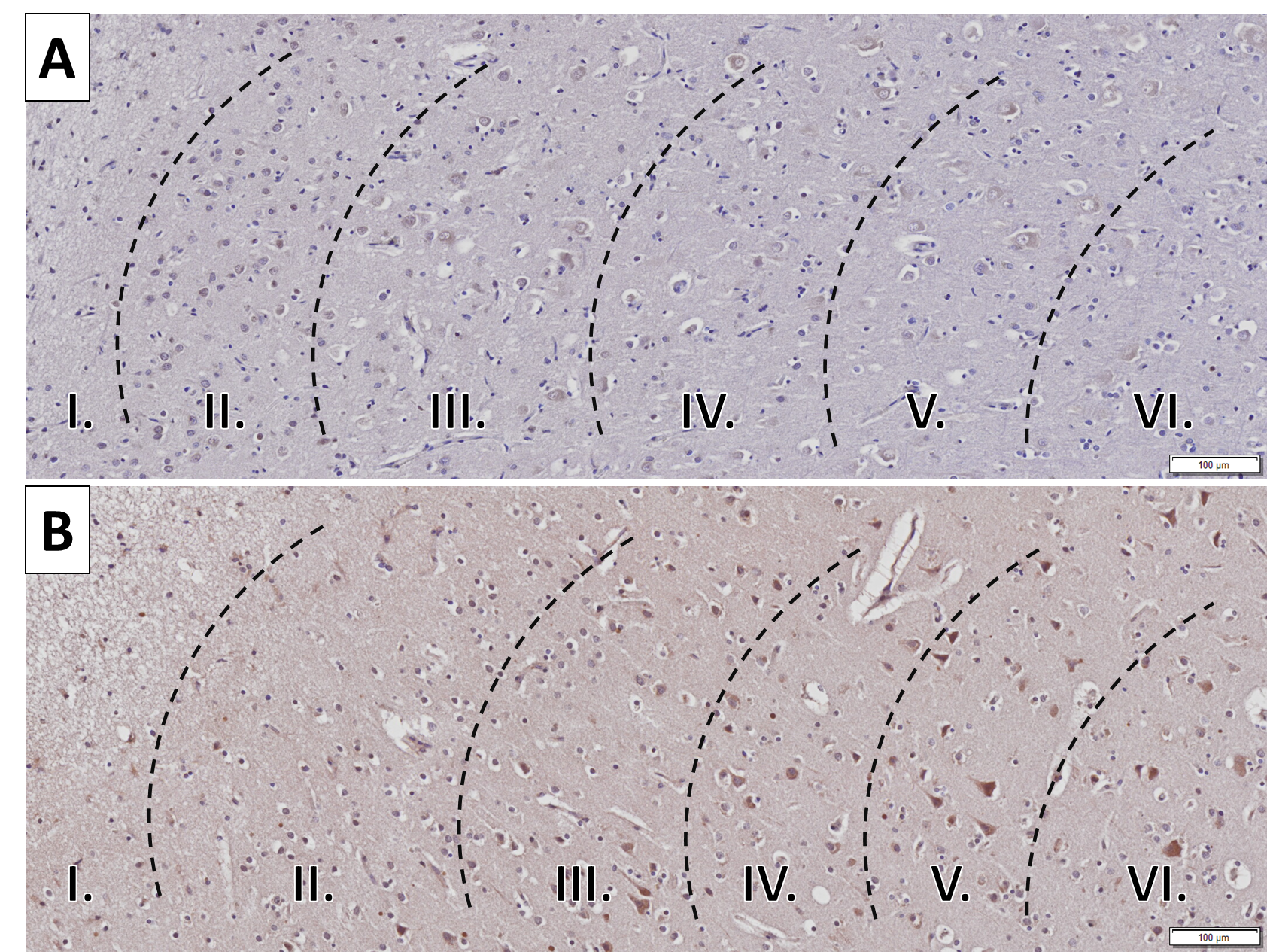


**Supplementary figure S2. Lemur tyrosine kinase 2 (LMTK2)-related mechanisms may contribute to neurodegenerative process in Alzheimer’s disease (AD).**

Under neuronal stress (e.g. β-amyloid accumulation, reactive oxygen species (ROS), etc.) increased calpain activation results in cleavage of p35 into p25 and p10. Cyclin-dependent kinase 5 (CDK5)/p25 complex has prolonged half-life/activity leading to hyperphosphorylation of downstream targets (e.g. tau protein). However, the role of CDK5/p25 complex in the activation of LMTK2 is unknown. Probably, alteration of upstream regulator and further neuronal stress-related mechanisms lead to reduced LMTK2 activity and expression. Disrupted LMTK2 signalling results in disinhibition on catalytic subunit of protein phosphatase 1 (PP1C) which in turn causes enhanced activity of Glycogen synthase kinase-3β (GSK3β). These changes are implicated in three neurodegenerative mechanism: i) Tau hyperphosphorylation by overactivated CDK5/p25 and GSK3β. ii) Dysregulated apoptosis by PP1 and GSK3β induced upregulation of proapoptotic Bcl-2-interacting mediator of cell death (Bim) and downregulation of antiapoptotic B-cell lymphoma-2 (Bcl-2) and B-cell lymphoma-extra-large (Bcl-xL) levels. iii) Disrupted axonal transport by GSK3β mediated phosphorylation of kinesin-1 light chain 2 (KLC2) leading to mothers against decapentaplegic homolog 2 (Smad2) cargo release. [White arrows=causal relation; black arrows=contribution; coloured arrow=direction of altered signalling pathway. Colour code: green=(over)activated enzymes; orange=disrupted/pathological processes].

*
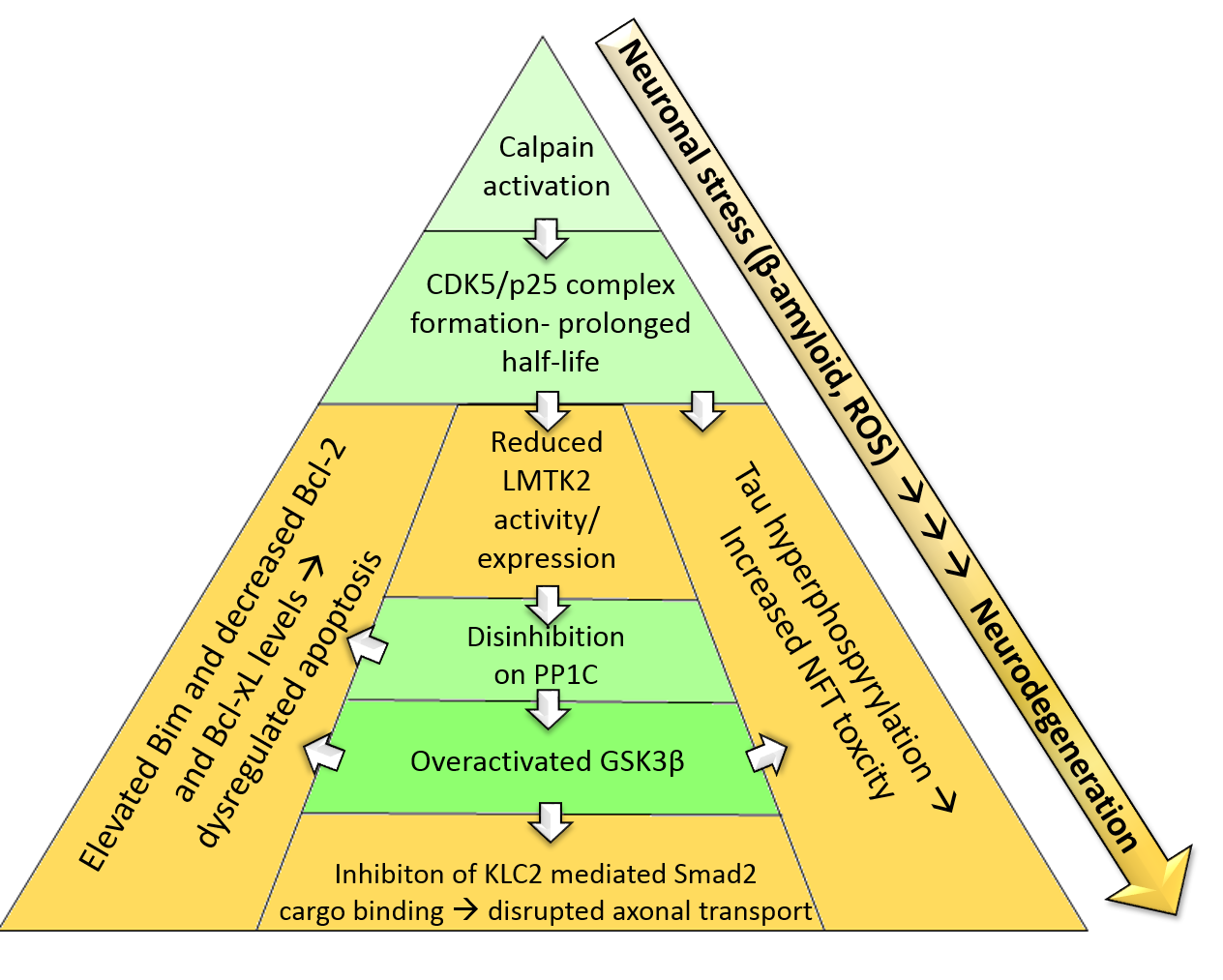
*
